# Contrasting morphometric responses to increasing urbanisation in congeneric sparrow species

**DOI:** 10.1038/s41598-024-67222-3

**Published:** 2024-07-13

**Authors:** Sage K. Naidoo, Dan Chamberlain, Chevonne Reynolds

**Affiliations:** 1 School of Animal, Plant and Environmental Sciences, University of the Witwatersrand, Private Bag 3, Johannesburg, 2050 South Africa; 2 Department of Life Sciences and Systems Biology, University of Turin, Via Accademia Albertina 13, 10123 Turin, Italy

**Keywords:** Africa, Bird ringing, Body size, Congeneric species, Indigenous, Non-native, Global south, Urban development, Ecology, Invasive species, Urban ecology

## Abstract

Increased urbanisation influences the morphometric traits of various species, often resulting in urban individuals being smaller than their non-urban counterparts. Urbanisation can affect fundamental eco-evolutionary patterns and impact species’ ability to adapt to and occupy rapidly changing environments through morphological changes. We investigated the morphometric responses of two passerine species, the non-native house sparrow (*Passer domesticus*) and its native congener, the Cape sparrow (*Passer melanurus*), along gradients of spatial and temporal urbanisation in South Africa over a 52-year period. The house sparrow was significantly heavier, larger and in better condition with increasing urban infrastructure and lower urban vegetation cover, while the Cape sparrow showed opposing trends along these gradients. Temporally, the house sparrow’s body mass increased consistently over the 52-year study period, suggesting changes in morphology were concomitant with increasing urbanisation over time. This study demonstrates distinct differences in the morphological responses of the non-native house sparrow and the native Cape sparrow to increasing urban development. These morphological responses may also underpin community-level changes caused by urbanisation, enhancing the capabilities of non-native species to thrive over their native counterparts in these environments.

## Introduction

Rapid urbanisation has accelerated changes to the biosphere, leading to the loss and homogenisation of global biodiversity^1,2^. Bird communities typically show reduced diversity and richness in urbanised areas^1,3–6^, with most species generally exhibiting lower nesting productivity and survival in urban areas^7,8^. Nevertheless, certain species demonstrate increased tolerance to urban habitats, adapting physiologically or behaviourally to exploit and thrive in urban ecosystems^9–13^.

Urban habitats tend to favour bird species that are better adapted to survive under novel conditions^12,14–16^. In particular, non-native species (i.e. species introduced to a novel environment where they establish viable populations) often have greater success in exploiting human-dominated areas^1,12,17^. Non-native species in urban habitats typically possess pre-existing adaptations that enable them to occupy these environments^12^. The establishment of these non-native, and generally invasive, bird populations has led to increased impacts on native species in urban environments, including increased competition for resources^18^, alterations to parasite regimes^19–21^, and increased risk of hybridisation^21–24^. These interactions have resulted in a heightened extinction risk for a range of primarily native bird taxa in urban ecosystems^25^.

The advantages non-native species have over native species may be a product of the influence of urbanisation on bird morphology, which is largely correlated to the effect of urban development on ecological niches and the novelty of anthropogenic landscapes^15,26,27^. Specifically, urban systems have been shown to cause changes to the size and condition of species^8,14,28,29^, with increased research effort now focusing on the mechanisms that underpin these patterns and responses^30,31^. Research on native urban bird populations has shown decreases in their body mass and body condition, and effects on other morphometric traits (i.e., tarsus, wing and tail length) in response to urbanisation^14,32,33^. However, there is a noticeable gap in understanding how these changes manifest in different geographical contexts, especially between the Global North and South^9^, which differ significantly in urban development patterns^34,35^.

The house sparrow (*Passer domesticus*; Linnaeus, 1758) has been the focus of multiple studies^14,28,29^ assessing the morphometric responses of bird populations to urbanisation in their native ranges because of its broad, cosmopolitan distribution^9^. Across its native urban distribution, the species has been found to show decreased size and body mass compared to non-urban populations^8,14,28,29^, while body condition has been reported to be either lowered^8,29^ or uninfluenced by urbanisation^32,36^. These patterns and responses generally do not account for the species in its introduced range. This suggests a potential gap in the assessment of global responses to urbanisation by bird species^9^, which may be influenced by differences in urban development between the house sparrows’ native and non-native ranges^6,34,35^. In South Africa, the non-native house sparrow is classified as an invasive species (NEMBA 2004), providing an opportunity to investigate the species’ responses to the urban environment within its introduced range. Additionally, the presence of a native congener, the Cape sparrow (*Passer melanurus*; Müller, 1776), offers a novel opportunity to compare the morphometric responses of a native and non-native congeneric pair under similar urban conditions. Furthermore, we bridge the geographic knowledge gap by assessing the morphometric responses of the house sparrow in its non-native range relative to previous studies that have focused mostly on its native ranges^14,28,29^.

There are three relevant hypotheses addressing the morphological responses of native and non-native sparrow species to urbanisation. First, the credit-card hypothesis^37^, which proposes a decrease in the body mass, body condition and body plan (i.e., concurrent and multidirectional changes across a bird’s wing, tarsus, and tail lengths) within increasingly urbanised systems. This hypothesis is strongly linked to decreased resource quality and the availability of novel food resources of lower nutritional quality (e.g., discarded food waste)^38^. These novel resources cause a reduction in individual fat reserves and subsequently lower reproductive output^37^. This hypothesis potentially explains observed decreases in the size of various morphometric traits of house sparrows within more urbanised systems in their native range^14,28,29^.

Second, Bergmann's rule^39^ predicts a thermoregulatory response associated with warmer climates that drives decreases in body size. Urban areas form urban heat islands (UHIs)^40^, which are consistently warmer than their surrounding habitats^41^. The invasion risk of mainly (sub-) tropical birds in Europe has been linked to the thermal niche of a species, which is influenced by morphological traits^42^. The morphological trait of body size is a critical determinant of metabolic rate, dispersal, and other life history traits^43,44^. Changes in body size resulting from urbanisation can have significant consequences for the structure and dynamics of ecological communities and may broadly affect urban ecosystem function^45^.

Third, the enemy release hypothesis^46^ posits that non-native species are released from the constraints to which native species are exposed, such as predators and parasites^20,28,47–49^. Species like the house sparrow, which are synurbic in nature, have the ability to exploit urban ecosystems more effectively^32,50^. Synurbic species may be less naïve to novel predation threats compared to native species^51^, either in terms of novel predators (e.g. cats and rats) or native predators adopting novel hunting behaviours in urban environments. Additionally, the release of non-native species from natural parasites may give them advantages in novel environments, which have different or less diverse parasite communities compared to the native range^20^. Predator-parasite or “enemy” release could thus correspond to an increased capability of these non-native individuals to exploit urban environments^48,49^, potentially driving a difference in their morphometric traits in the introduced range.

These three hypotheses lead to different predictions in terms of the response of morphological traits to urbanisation between native and non-native species. If either the credit-card hypothesis or Bergmann's rule were the dominant mechanism explaining changes in the morphometrics of urban birds, we would expect non-native and native species to respond similarly. The enemy release hypothesis on the other hand predicts positive effects of urbanisation in non-native species and negative effects in native species.

We used long-term morphometric data from the South African bird ringing scheme (SAFRING) to quantify changes in morphological traits in response to urbanisation in the non-native house sparrow, as a known urban exploiter, and its native congener, the Cape sparrow, to determine whether the response differs between the native and non-native species. Specifically, we assessed the relationship between urban cover, in terms of both artificial surfaces and urban vegetation cover, and the species’ morphometrics in terms of body mass, body condition and body plan. Furthermore, we assessed how species morphometrics varied over a 52-year period of increasing urbanisation in South Africa.

## Methods

### Morphometric data and study species selection

**Figure 1:**
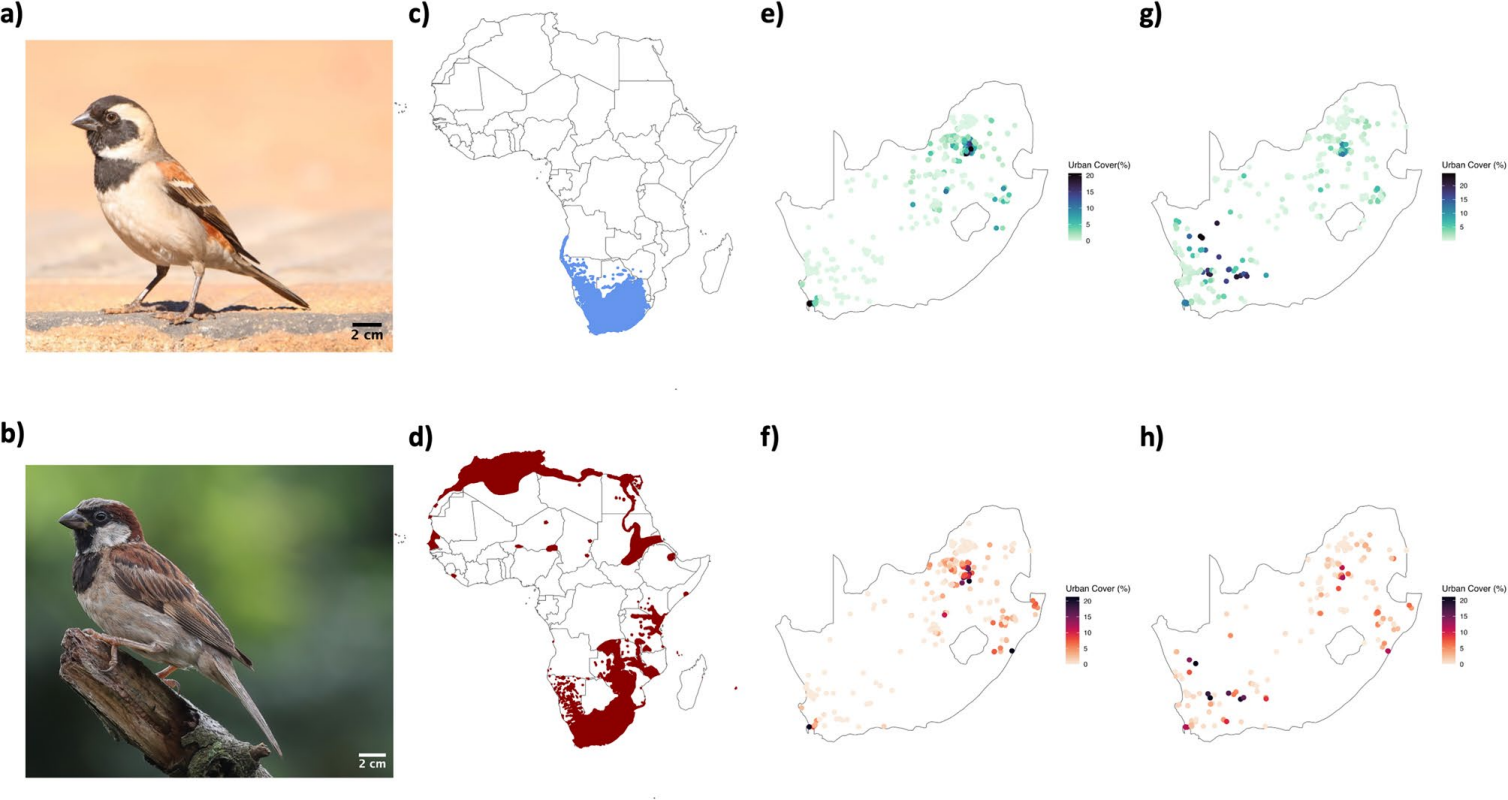
Images of the two congeneric study species: (**a**) the Cape sparrow (*Passer melanurus*), native to southern Africa, and (**b**) the house sparrow (*Passer domesticus*), a widespread and adaptable bird found in urban and rural areas worldwide. The distribution of Cape (**c**) and house (**d**) sparrows within Africa, and the locations (**e**–**h**) where individual sparrows were ringed within South Africa are displayed. The colour represents the differences in measures of overall urban cover extracted within a 10 km radius of each ringing location for Cape sparrows relative to the 1990 (**e**) and 2020 (**g**) South African Land Cover datasets, and house sparrows relative to the 1990 (**f**) and 2020 (**h**) South African Land Cover dataset. The two different time periods for the land cover datasets were chosen to control for changes in urban land cover over the 52-year period and were matched as closely as possible to the ringing date for each individual record. Photographs provided by Christopher Shortland (**a**) and Dominic Rollinson (**b**).

Morphometric data on the Cape sparrow (Fig. 1a), a native South African species, and the house sparrow (Fig. 1b), a non-native and actually invasive species (classified under Category 3 of NEMBA 2004), were acquired from South Africa’s dedicated bird ringing initiative, SAFRING. A ringing initiative, such as SAFRING, involves tracking individual birds through unique numbered rings to monitor movements and collect morphological and demographic data. The SAFRING dataset used here spans from 1970 to 2021 and includes records of morphometric and other biometric characteristics of the two sparrow species across South Africa^52^. These data were collected by various professional ornithologists and amateur citizen scientist bird ringers at approximately 1000 ringing locations in the country, following methods and procedures outlined in the SAFRING Bird Ringing Manual^52^.

The morphometric trait data for individual birds generally included measures of body mass (in grams), which was the most frequently recorded trait for both species. Other measured traits included the length (in mm) of the tarsus, wing, culmen, head and tail (Supplementary Table 1), although the recording of these specific measures varied among individual ringers and ringing locations. In this study, all recorded traits were included except those removed during our pre-analysis screening (see below—Data analysis).

The Cape sparrow, with an average body mass of 29.4 g (males) and 29.6 g (females), is near-endemic to southern Africa (Fig. 1c) and found throughout the region in grasslands, savannas, shrublands and woodlands. It is highly urban-tolerant and commonly found in agricultural fields and surrounding homesteads, residential gardens, and urban green spaces^53,54^. The house sparrow, introduced from Europe and the Middle East to southern Africa in the early 1900s^54,55^, is only slightly smaller, with an average mass of 25.4 g (males) and 26.2 g (females) in the South African population^54^. The house sparrow has a broad distribution in its native range, from boreal regions in the north to semi-desert habitats in the south. Within South Africa, the house sparrow’s geographic range nearly perfectly overlaps with that of the Cape Sparrow (Fig. 1d). Thus, the two species experience similar habitat and climatic ranges, although the synurbic nature^32,50^ of the house sparrow might account for a slightly wider distribution throughout highly urbanised systems^53,54^.

The diet of both species is very similar, as both generally consume nectar, fruit, seeds and insects (including ants and termites)^53^. While both species build untidy ball-shaped nests of grass, sticks, and fibres, and tend to form monogamous pairs and nest colonially, their specific reproductive and breeding patterns differ slightly^53^. The Cape sparrow produces a clutch of 2–6 eggs seasonally, while the house sparrow produces clutches of 1–6 eggs throughout its year-long laying-cycle in the South African population^53^.

### Study site selection

Morphometric data were available across the entire extent of South Africa, including areas of high and low urban cover, as well as more rural regions. South Africa’s urban development has been strongly influenced by the end of the Apartheid regime and the subsequent growth in the human population, which increased by 49.3% between 1996 and 2022, with an annual increase of 1.9% (Statistics SA). This population growth coincided with migration into cities^56,57^, thereby increasing levels of urban development (Statistics SA).

To analyse the changes in urban cover over time, we used two separate land cover datasets from 1990 and 2020, obtained from the South African National Land Cover (SANLC) database. These two datasets allowed us to account for the approximately 50-year temporal span of the ringing data and to assign these ringing data to each corresponding period (see below). By doing so, we ensured that each ringing record was matched as closely as possible to historic (1990) or contemporary (2020) land cover.

The SANLC datasets categorise the country into its dominant land cover classes using Landsat 8 imagery with a resolution of 30 m by 30 m (SANLC). Four metrics of urban cover were extracted from each dataset. For both the 1990 and 2020 land cover datasets, we produced an overall representative measure of urban land cover in South Africa (range = 0.00–25.0%). We also extracted three sub-classes of urban cover: urban infrastructure (range = 0.0–50.0%), which includes only impervious or built surfaces within formal and informal settlements, commercial and industrial areas, and roadways; and two measures of urban vegetation cover-grass [range = 0.0–25.0%] and woody [range = 0.0–8.0%] vegetation cover).

To best match the morphometric trait measures to the extracted land cover metrics for 1990 and 2020, we divided the morphometric trait dataset into two periods: 1970 to 2005 and 2006 to 2021. Although land cover data were not available prior to 1990, the rate of urbanisation, in terms of human population growth, was relatively low before this year (Urban population trends). Thus, data from 1970 to 2005 were assumed to be representative of earlier periods. This assumption was largely due to the apartheid regime^58^, which limited certain populations to ‘homeland’ or township areas beyond the urban centres^57,59^. Urban sprawl only increased post-apartheid, both in terms of built infrastructure and population sizes in urban regions^57,59^.

Using the ringing location of each individual sparrow (Fig. 1e–h) assigned to either the historic or contemporary period, we extracted the percentage cover of all four urban cover metrics from the land cover datasets within a 10 km radius buffer. All extractions were performed in Google Earth Engine^60^. The percentage urban cover for each metric was calculated as the pixel area of each individual urban cover type divided by the total land cover areas within the 10 km radius zone. This method allowed us to correlate the morphometric data with the corresponding urban cover metrics for the respective periods.

### Climatic and vegetation productivity variables

We extracted measures of average annual minimum temperature and precipitation, and the annual Normalised Difference Vegetation Index (NDVI) for each year between 1990 and 2021 within the same 10 km buffer of each ringing location using Google Earth Engine’s TerraClimate^60^ and MOD13A2.061 band^61^, respectively. The change in temperature between this period did not vary greatly (average 1990 = 16.12 ± 2.64 °C and average 2021 = 16.79± 2.81 °C). However, to account for the potential influence of these climate variables across space, we used the average measures of the climate data for the span of our land cover temporal range. These measures provided important environmental controls to account for their influence on avian morphometrics, in particular in relation to Bergmann’s Rule, which may also be in effect across South Africa’s varied climatic zones^33,39^.

### Temporal analysis

We used body mass (the trait with the most data) to assess if there was an additional temporal influence on the morphometric trends of these two sparrow species over the approximately 50-year span. Measures of body mass were assessed within the most densely urbanised regions (> 10% overall urban cover). These measures were only extracted for the Gauteng and Western Cape provinces as the other provinces had too few highly urbanised regions within the range of SAFRING ringing locations to produce meaningful results for the temporal analysis.

### Statistical analysis

Variability in citizen science and bird ringing practices can create messy data^62,63^. In this study, variability in measurement techniques between individual ringers may introduce measurement bias. To account for this, datasets underwent thorough screening, taking a conservative approach to eliminate likely errors. For each species, we calculated the overall average of each morphometric trait to exclude outliers, identified as trait measures that substantially diverged from the calculated, and previously reported^53^, average measures of each specific trait. We then used the 95% confidence interval of the mean for each trait to further remove individuals that skewed the normality of the datasets, discarding approximately 14% of the original data. Records that were not identified as adults or those that were not sexed were removed, as well as individuals ringed and measured in the Limpopo and Eastern Cape provinces, where too few trait measures were recorded. The final dataset included 26,587 points across both species, with the Cape sparrow accounting for 21,064 points and the house sparrow 5272 points.

To explore the effect of urban infrastructure, urban vegetation cover, and overall urban cover on species morphology, we used generalised linear mixed effects models^64^. Body mass, body condition, and body plan were the three main response variables in the models, with individual morphometric traits (e.g., tarsus and wing length) analysed in a supplementary analysis. Body mass measures were extracted from the SAFRING datasets, while body condition was expressed as an index calculated by dividing body mass by the tarsus length, a commonly used measure of body condition^65^. A metric for body plan, representing overall changes in morphology, was generated through Principal Component Analysis (PCA). We used a PCA to constrain the measures of body mass, and tarsus, wing, culmen, head and tail lengths, to a single axis, PC1. This individual component explained 46% of the overall variance and was used as a representation of the overall body plan (Supplementary Table 3). All variables had positive loadings on PC1, with head length (0.77) and wing length (0.75) having the highest individual loadings (Supplementary Table 3). Note that the sample size was reduced for this analysis due to missing data for some of the morphometric traits (n = 1351).

For each response variable (body mass [n_Cape_ = 21,064; n_House_ = 5272], body condition [n_Cape_ = 3151; n_House_ = 53]) and body plan [n_Cape_ = 956; n_House_ = 394] as expressed by PC1), we included either urban infrastructure, urban vegetation cover (grass and woody vegetation), or overall urban cover, along with species (as a two-level factor) and the interaction between species and the relevant land cover variables as fixed effects (see Supplementary Table 2 for model formulations). We also incorporated climate variables, sex and the season in which the sparrows were ringed as fixed effects, the latter represented by a conversion of the ringing month into a circular measure expressed in radians to account for seasonal continuity. By incorporating these variables into the model, we accounted for known influences of sex^66,67^ and seasonal fluctuations^28^ on the traits of the sparrows. We also fitted ringer identity and ringing location as crossed random effects (note that individual ringers often operate in different ringing locations) to account for likely individual- and site-level effects on morphometrics.

The above models were repeated for the other traits (tarsus [n = 2853], wing [n = 16,186], tail [n = 7388], culmen [n = 4305] and head [n = 2872] lengths). The outputs (model 13–32 in Supplementary Table 2; Supplementary Table 7–11) were used to create a conceptual representation of how the overall body plan of each species changed along the urban infrastructure, vegetation and overall urban cover gradients. A model was also fitted to assess the long-term temporal influence on body mass (as it had the largest sample size [n_Cape_ = 14,397, n_House_ = 2979]) relative to the changes in the temporal gradient. In this model, we included an interaction between species and year to assess if there was a difference between species over time, as well as the sex, season, and climate variables as fixed effects and ringer identity and site as random effects as previously described (model 33 in Supplementary Table 2).

NDVI was found to be highly correlated with precipitation (r = 0.86) and was thus excluded from the models. There was no evidence of collinearity amongst the other continuous fixed effects (r < 0.5). All variables were scaled and centred prior to the analysis (our graphs of model outputs are presented on the original scale to facilitate interpretation). Model residuals and diagnostic plots were used to ensure data were a good fit. We also ran models using the log of each response variable and found results to be very similar, hence all models were produced using the untransformed data to facilitate interpretability.

All analyses were conducted in R version 4.2.0—“Vigorous Callisthenics”^68^, with the linear-mixed effects models run in the ‘lme4’ package^64^.

## Results

### Body mass and body condition

**Figure 2:**
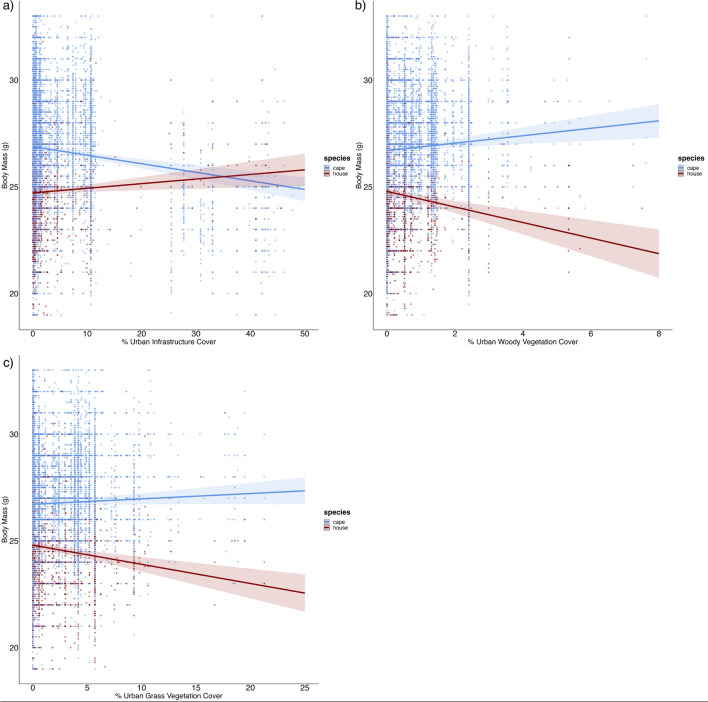
Changes in the body mass of Cape sparrows (blue; n = 21,064) and house sparrows (red; n = 5272) were analysed along different urban gradients in South Africa. The interaction of body mass responses between the two species was examined in response to  (**a**) increased urban infrastructure, (**b**) increased urban woody vegetation cover, (**c**) increased urban grass cover. Body condition showed similar trends (Supplementary Fig. 1).

For body mass, there was a significant interaction between sparrow species and both urban infrastructure (*p* < 0.001) and urban vegetation cover (grass cover [*p* < 0.001] and woody cover [*p* < 0.001]), indicating that the two species responded differently to these urban gradients (model 1–4 in Supplementary Table 2; Supplementary Table 4). Body mass increased in the house sparrow and decreased in the Cape sparrow with increasing urban infrastructure (Fig. 2a). These trends were reversed for both urban vegetation gradients (Fig. 2b, c). There was no significant interaction between sparrow species and overall urban cover (Supplementary Table 4). Body condition showed similar responses (i.e., significant interactions) to urban infrastructure (*p* = 0.01) and woody cover (*p* = 0.01; Supplementary Fig. 1; model 5–8 in Supplementary Table 2; Supplementary Table 5). There was no significant interaction between species and overall urban cover and grass cover (Supplementary Table 5).

### Body plan

**Figure 3:**
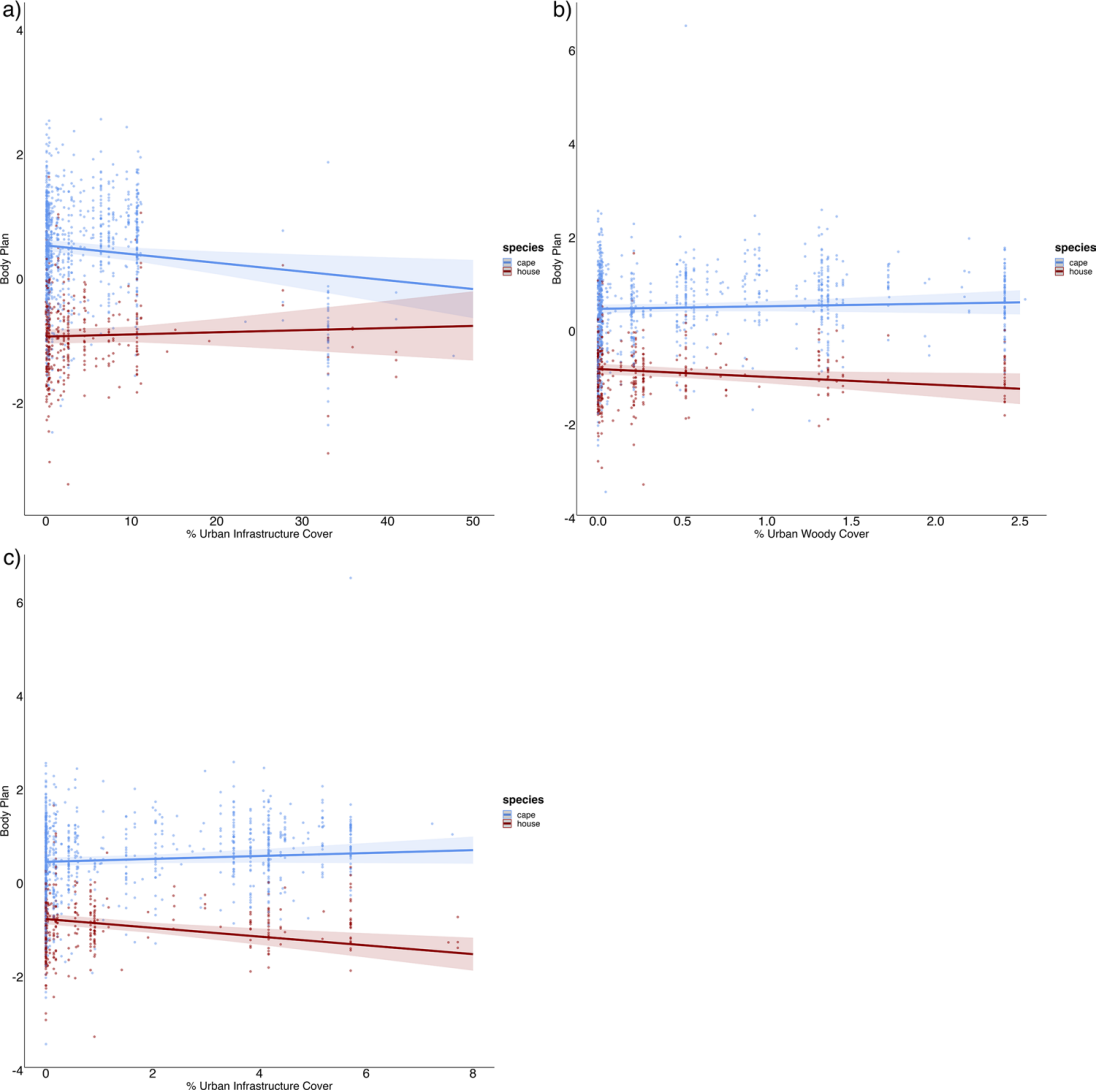
Changes in the body plan of Cape sparrows (blue, n = 956) and house sparrows (red, n = 394) are represented along the different urban gradients. The interaction of body plan responses between the two sparrows was examined in response to (**a**) an increase in the urban infrastructure cover, (**b**) an increase in urban woody vegetation cover, (**c**) an increase in the urban grass vegetation cover.

Body plan (i.e., PC1, our representation of overall morphology) showed significant interactions between species and urban infrastructure (*p* = 0.004), urban woody cover (*p* < 0.001) and urban grass cover (*p* < 0.001), indicating that the two species responded differently to these urban gradients (model 9–12 in Supplementary Table 2; Supplementary Table 6). The Cape sparrow showed a negative relationship and change in body plan with increasing urban infrastructure, while the house sparrow showed a positive relationship (Fig. 3a). These trends were reversed for both urban vegetation gradients (Fig. 3b, c).

**Figure 4:**
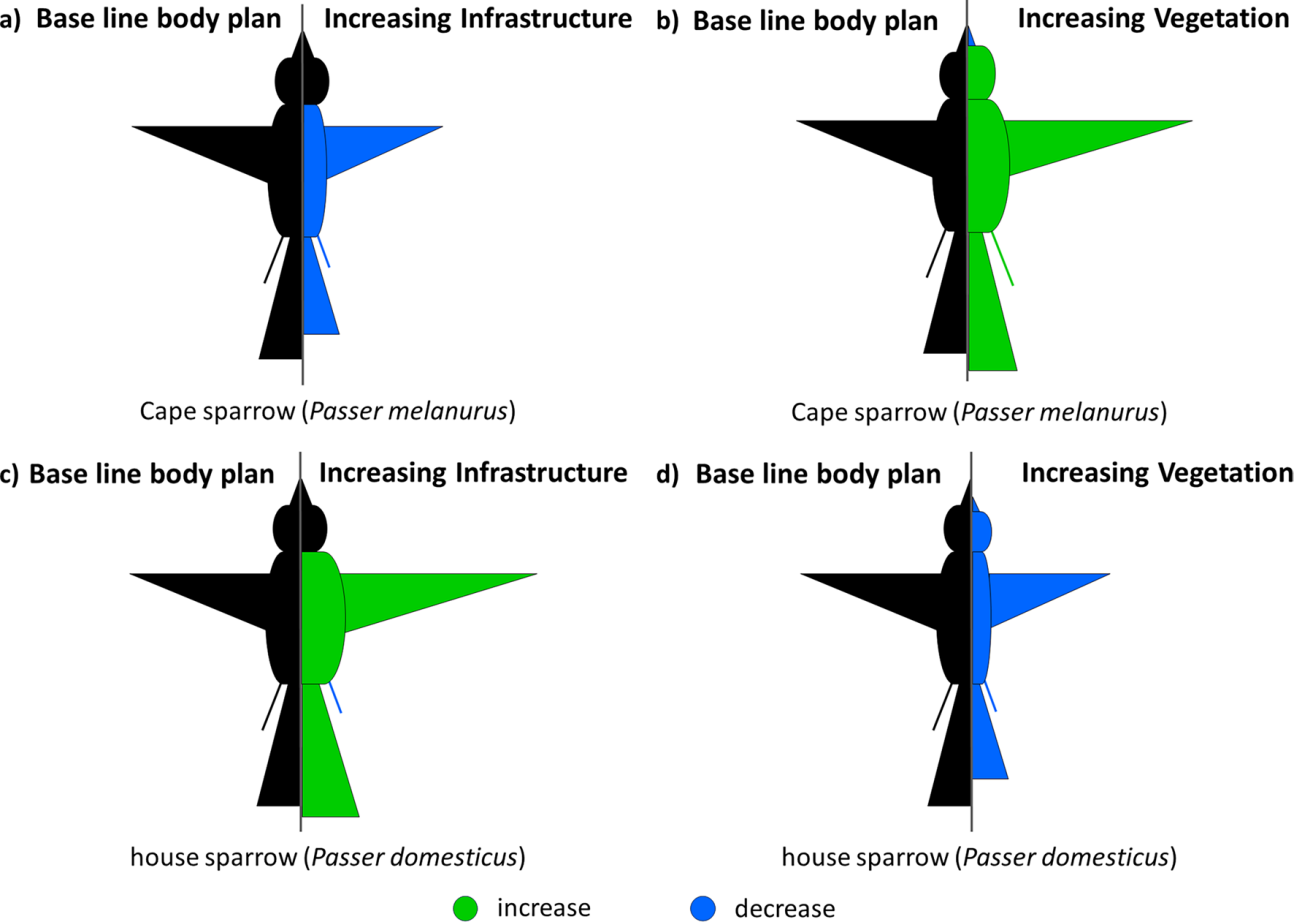
Conceptual diagram of the observed changes in morphometric traits of the Cape and house sparrow. Measures of body mass (n = 26,336) and overall size (represented by the belly), tarsus length (n = 2853), wing length (n = 16,186), culmen length (n = 4305), head length (n = 2872) and tail length (n = 7388) of the sparrows are presented relative to a baseline body plan (in black). Changes in body traits of the Cape sparrow are shown relative to (**a**) the increasing urban infrastructure gradient and (**b**) the increasing urban vegetation gradient. Changes in body triats of the house sparrow are shown relative to (**c**) the increasing urban infrastructure gradient and (**d**) the increasing urban vegetation gradient.

When considering all morphological traits independently, the two species exhibited differing morphological responses to urban infrastructure and urban vegetation cover, as demonstrated by significant interactions (model 13–32 in Supplementary Table 2; Supplementary Table 7–11). The Cape sparrow showed a decrease in its tarsus, wing and tail length with increased urban infrastructure (Fig. 4a) but exhibited reversed patterns with increased urban vegetation (both urban grass and woody cover combined, Fig. 4b). Conversely, the house sparrow showed increases in wing and tail length increased with increasing urban infrastructure (Fig. 4c) but decreases with increasing urban vegetation cover (Fig. 4d).

### Changes along the temporal gradient

**Figure 5:**
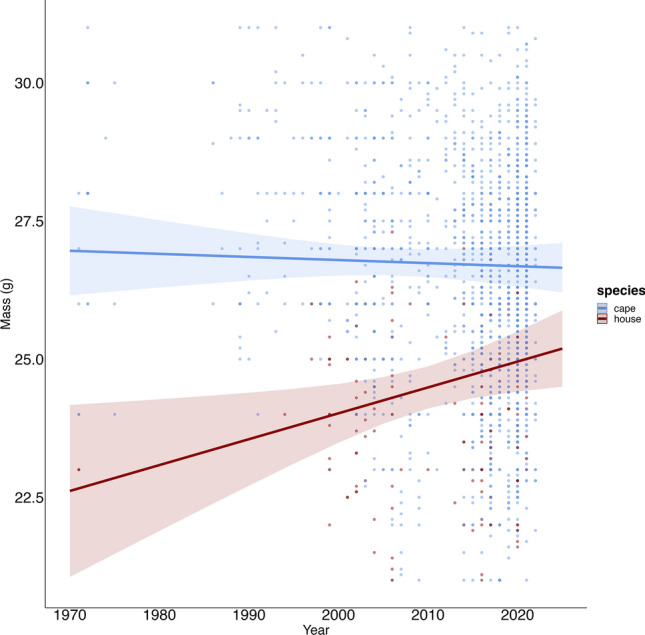
The relationship between the changes in the body mass of Cape sparrows (blue, n = 14,397) and house sparrows (red, n = 2979)  in highly urban settings was assessed over a 50-year period. This relationship was analysed in relation to the year each individual sparrow was ringed.

There was a significant interaction between year and species, demonstrating different temporal trends in body mass between the two species. Specifically, the Cape sparrow showed a slight decrease in body mass over time, while the house sparrow showed a strong increase (Fig. 5; model 33 in Supplementary Table 2). These changes occurred over a period of marked urbanisation in South Africa, with total urban cover more than doubling from 1.86% in 1990 to 3.81% in 2020 (SANLC).

## Discussion

Urbanisation had a strong effect on the morphology of the two sparrow species, but their responses were generally opposing. Increased urban infrastructure positively influenced the overall morphometric responses of the non-native house sparrow, while it generally had a negative influence on the native Cape sparrow. These differing responses may relate to their native or non-native provenance. The Cape sparrow conformed to the expected morphometric responses of birds within their native urban ranges^28,29,36^, becoming smaller with increasing urban cover but increasing in size with more vegetation cover, suggesting an association with less urbanised systems. Conversely, the house sparrow increased in size and condition along the urban infrastructure gradient, showing the opposite effect to that observed within its native range^14,32^. Our study thus supports the idea that, as a non-native species, highly urbanised systems in South Africa favour the house sparrows’ development, compared to more vegetated systems where its morphometric responses align with patterns from its native range.

The results of our study show support for the enemy release or predator-parasite release hypothesis, as the two species showed different responses to the same urban infrastructure gradient. Release from the house sparrow’s natural predators^28^ and parasites^20^ may be the underlying driver of their morphometric responses, at least within highly urbanised landscapes. While the natural predators of the species may be similar to that of the Cape sparrow (e.g., domestic cats and sparrowhawks), several factors may reduce the house sparrow's susceptibility to predation. For example, reduced naivety towards synanthropic predators^51^ because of its largely synurbic nature ^32,50^. Sih et al.^51^ observed greater susceptibility of native species to predation in novel environments compared to species that are more accustomed to these novel conditions. As such, the house sparrow may be less susceptible to predators in these urban spaces. Additionally, within urban areas, species that are more “urban-tolerant” tend to be less affected by parasites prevalent in urban environments^20^, which impact native populations^47^. House sparrows have been shown to be “released” from parasites in their non-native ranges, with a lower quantity and diversity of parasites compared to their native range^20^. The non-native house sparrow is thus more likely to resist infection and possibly be in better condition ^47,69,70^ than the native Cape sparrow, which carries a heavier parasite load.

Our overall results support the enemy release hypothesis. Nevertheless, both resource quality and availability (i.e., the credit-card hypothesis)^37^ and thermoregulatory responses (i.e., Bergmann’s rule)^39^ may play a role in the response of the Cape Sparrow to urbanisation. The smaller size of the Cape sparrows in increasingly urbanised systems suggests that the lower quality of resources^38^ and warmer urban climates induce physiological and thermoregulatory stresses linked to their water use and energy reserves^71^. Reduced mass and size may help combat these constraints in highly urbanised areas, whereas increased urban vegetation may buffer the effects, resulting in larger Cape sparrows within more vegetated urban systems^72^. More research on resource availability in urban landscapes and the physiology of organisms in these varied landscapes is needed to fully disentangle these two hypotheses.

Temporal trends in the body mass of the two species over a period of increasing urbanisation showed the house sparrow strongly increasing in mass over time, while the Cape sparrow exhibited modest decreases. These trends further support the assertion that urbanisation causes an increase in habitat quality for the house sparrow and, to a lesser extent, diminishes it for the Cape sparrow. While the two species do co-exist in these habitats, their responses may differ because of urban effects on habitat quality for each species. Whether such temporal changes represent adaptations or plastic responses to environmental change is subject to debate^73,74^. There is evidence in other species for a genetic basis to morphometric responses to drivers associated with urbanisation^75^. For example, in their native ranges in Israel^76^ and England^77^, house sparrows have shown decreases in body mass over time, attributed to a thermoregulatory adaptive response driven by climate change. Such a response can be discounted in the non-native range in South Africa, where body mass and body size were greater in urban areas and where these traits increased over time, suggesting a selective advantage of being larger. Thus, significant changes in the body size of house sparrows over time in South Africa may reflect adaptive evolution in response to a novel environment. Larger body sizes initially resulting from enemy release may provide an additional competitive edge to non-native species in urban environments, allowing them to better cope with highly fragmented urbanised landscapes where larger body size can be linked to increased dispersal ability^44,45^. House sparrows also showed longer wing lengths in more urban landscapes (Fig. 4; model 17–20 in Supplementary Table 2; Supplementary Table 8), providing further evidence that improved dispersal ability may be a key driver of morphometric shifts in urban ecosystems.

In the house sparrow’s native range, many urban populations are in decline^78,79^. There have even been declines in its non-native range in North America^80^, and in South Africa, a comparison between the first and second South African Bird Atlas Projects (SABAP1 [1987–1992] and SABAP2 [2007-ongoing]), shows an approximate 12% reduction in the reporting rate of the species for the most urbanised province, Gauteng^81^. These trends seem at odds with our findings, which suggest urban areas represent good quality habitats for non-native house sparrows in South Africa. The explanation may be related to the degree of wider urban economic development, where extensive and intensive human settlements filter out even the most competitive urban species by altering resource regimes^82^. Declines in house sparrow populations are particularly well-documented in wealthier countries of the Global North, where changes to the urban habitat linked to economic development may be having detrimental effects on the species^79^. Thus, despite the inference that house sparrows are thriving in urban landscapes in South Africa based on their morphometric trends, it is possible that South African cities are now also reaching a pivotal point in their more recent socio-economic development^34^, where even populations of competitive and non-native species are declining. This suggests a possible non-linear relationship between house sparrow population trajectories, adaptation, and urban economic development, which would be worth exploring by comparing house sparrow populations from different socioeconomic contexts.

## Limitations

We have interpreted the contrasting morphometric responses of Cape and house sparrow as reflecting differences in urban habitat quality. However, although bird size and other condition indices are often used as proxies for individual quality^65^, they may not always be a true measure of success in these environments as they don’t reflect improved fitness. Nevertheless, our data do show that house sparrow body sizes have increased steadily through time, suggesting the heritability of this phenotype and probable fitness benefits of being larger in more urbanised environments. What is less apparent is whether house sparrow quality reflects improved habitat quality, or rather just the ability of this species to better exploit marginal habitat due to its non-native provenance. For Cape sparrows, bird quality is even more difficult to interpret, as changes in body size appear to be less marked through time.

Urban environments can introduce a range of novel ecological pressures, such as increased predation and disturbance, which might lead to changes in bird body size, and which are not solely indicative of habitat quality. Factors such as behaviour, physiology and genetics play a pivotal role in determining how species cope with urbanisation and its challenges^71,83,84^. Thus, while trends in sparrow size and other morphological traits have provided valuable insights into how native and non-native species respond to increased levels of urbanisation, experimental work on habitat quality and avian adaptation, including behavioural responses to predators in urban environments, is necessary to determine the likely mechanisms underpinning these responses.

## Conclusion

Our study demonstrates distinct morphological responses between a native and a non-native congeneric pair along an urbanisation gradient. These findings highlight the potential for diverse and novel impacts of ongoing urbanisation on both native and non-native species, which could precipitate community-level shifts in urban wildlife. As urbanisation intensifies, careful consideration of the implications for both native and non-native species is needed in urban contexts. Urban conservation and sustainable development should prioritise making urban centres more accessible and suitable for native species, as they are disproportionately negatively impacted by urban development^1,85^. Our study shows the positive effects of urban green infrastructure on a native species’ “quality”, suggesting that well-managed green spaces hold promise as conservation interventions in urban landscapes^86^. This may be particularly relevant for Global South countries like South Africa, where urban green space planning for biodiversity conservation is limited^86^.

However, the intricacies of these responses are far from fully understood. Future research should investigate the influence of socio-economic factors on urban bird morphology, especially in the developing world where strong socio-economic gradients can supersede ecological factors in their influence on biological communities^63,87,88^. Furthermore, comparing the morphological and population trends between Global North and Global South cities will improve our understanding of global-scale patterns and the long-term effects of urbanisation on avian biology^6^. Through better understanding of these complexities, we can begin to inform urban planning strategies that foster coexistence between humans and the rich avian diversity that inhabits our cities.

### Supplementary Information

Supplementary Information.

## Data Availability

Morphometric data is an open access dataset available through SAFRING (SAFRING). All other data is open access available through Google Earth Engine (Google Earth Engine).
