# Supplementary material for: Contrasting morphometric responses to increasing urbanisation in congeneric sparrow species
Source: Sci Rep. 2024 Jul 13;14:16170. doi: 10.1038/s41598-024-67222-3 (PMC11246440; doi:10.1038/s41598-024-67222-3)
Supplement: Supplementary file 1 — Supplementary Information. [file 41598_2024_67222_MOESM1_ESM.pdf]

Contrasting morphometric responses to increasing urbanisation in congeneric native and invasive sparrow species

Sage K. Naidoo, Dan Chamberlain and Chevonne Reynolds

Supplementary materials

Supplementary Table 1: Summary details of the SAFRING morphometric trait data, based on the combined measures of the Cape and house sparrows (descriptions derived from the SAFRING bird ringing manual (de Beer *et al*. 2001)

**Trait**	**Description**	**Mean**	**Standard Error**	**Sample Size (n)**
**Body mass**	Mass of the bird subtracted from the mass of the bird in its' carrying bag.	26.42	0.02	26,336
**Tarsus length**	Length of the tarsometatarsal bone	19.78	0.03	2,853
**Wing length**	Distance on the closed wing from the foremost extremity of the carpus to the tip of the longest primary feather	77.47	0.03	16,186
**Culmen length**	Distance from the bill tip to either the cere, feathering, or union with the skull	12.75	0.01	4,305

**Head length**	Distance from the back of the head to the tip of the bill	31.75	0.01		2,827
**Tail length**	Distance from the root of the central pair of tail feathers to the tip of the longest feather	59.32	0.04		7,388

Supplementary Table 2: Details of the statistical models used in the data analysis.

**Model**	**Type**	**Dependent Variable**	**Explanatory Variable**	**Fixed Effects**	**Random Effects**	**n**
**1**	lmer	Body Mass	Urban_Overall*species	Sex, Season, Temperature, Precipitation	Location, Ringer	26,336
**2**	lmer	Body Mass	Infrastructure*species	Sex, Season, Temperature, Precipitation	Location, Ringer	26,336
**3**	lmer	Body Mass	Woody_Cover*species	Sex, Season, Temperature, Precipitation	Location, Ringer	26,336
**4**	lmer	Body Mass	Grass_Cover*species	Sex, Season, Temperature, Precipitation	Location, Ringer	26,336
**5**	lmer	Body Condition	Urban_Overall*species	Sex, Season, Temperature, Precipitation	Location, Ringer	3,687

**6**	lmer	Body Condition	Infrastructure*species	Sex, Season, Temperature, Precipitation	Location, Ringer	3,687
**7**	lmer	Body Condition	Woody_Cover*species	Sex, Season, Temperature, Precipitation	Location, Ringer	3,687
**8**	lmer	Body Condition	Grass_Cover*species	Sex, Season, Temperature, Precipitation	Location, Ringer	3,687
**9**	lmer	Body Shape	Urban_Overall*species	Sex, Season, Temperature, Precipitation	Location, Ringer	1,351
**10**	lmer	Body Shape	Infrastructure*species	Sex, Season, Temperature, Precipitation	Location, Ringer	1,351
**11**	lmer	Body Shape	Woody_Cover*species	Sex, Season, Temperature, Precipitation	Location, Ringer	1,351
**12**	lmer	Body Shape	Grass_Cover*species	Sex, Season, Temperature, Precipitation	Location, Ringer	1,351
**13**	lmer	Tarsus Length	Urban_Overall*species	Sex, Season, Temperature, Precipitation	Location, Ringer	2,542
**14**	lmer	Tarsus Length	Infrastructure*species	Sex, Season, Temperature, Precipitation	Location, Ringer	2,542

**15**	lmer	Tarsus Length	Woody_Cover*species	Sex, Season, Temperature, Precipitation	Location, Ringer	2,542
**16**	lmer	Tarsus Length	Grass_Cover*species	Sex, Season, Temperature, Precipitation	Location, Ringer	2,542
**17**	lmer	Wing Length	Urban_Overall*species	Sex, Season, Temperature, Precipitation	Location, Ringer	16,186
**18**	lmer	Wing Length	Infrastructure*species	Sex, Season, Temperature, Precipitation	Location, Ringer	16,186
**19**	lmer	Wing Length	Woody_Cover*species	Sex, Season, Temperature, Precipitation	Location, Ringer	16,186
**20**	lmer	Wing Length	Grass_Cover*species	Sex, Season, Temperature, Precipitation	Location, Ringer	16,186
**21**	lmer	Culmen Length	Urban_Overall*species	Sex, Season, Temperature, Precipitation	Location, Ringer	4,305
**22**	lmer	Culmen Length	Infrastructure*species	Sex, Season, Temperature, Precipitation	Location, Ringer	4,305
**23**	lmer	Culmen Length	Woody_Cover*species	Sex, Season, Temperature, Precipitation	Location, Ringer	4,305

**24**	lmer	Culmen Length	Grass_Cover*species	Sex, Season, Temperature, Precipitation	Location, Ringer	4,305
**25**	lmer	Head Length	Urban_Overall*species	Sex, Season, Temperature, Precipitation	Location, Ringer	2,827
**26**	lmer	Head Length	Infrastructure*species	Sex, Season, Temperature, Precipitation	Location, Ringer	2,827
**27**	lmer	Head Length	Woody_Cover*species	Sex, Season, Temperature, Precipitation	Location, Ringer	2,827
**28**	lmer	Head Length	Grass_Cover*species	Sex, Season, Temperature, Precipitation	Location, Ringer	2,827
**29**	lmer	Tail Length	Urban_Overall*species	Sex, Season, Temperature, Precipitation	Location, Ringer	7,388
**30**	lmer	Tail Length	Infrastructure*species	Sex, Season, Temperature, Precipitation	Location, Ringer	7,388
**31**	lmer	Tail Length	Woody_Cover*species	Sex, Season, Temperature, Precipitation	Location, Ringer	7,388
**32**	lmer	Tail Length	Grass_Cover*species	Sex, Season, Temperature, Precipitation	Location, Ringer	7,388

**33**	lmer	Body Mass	Year*species	Sex, Season, Temperature, Precipitation	Location, Ringer	17,376

Supplementary Table 3: Percentage loadings generated from the Principal Component Analysis for PC1 which integrated measures of the body mass, and lengths of the wing, culmen, head, tail, and tarsus, into a single measure.

	**PC1**
**Body Mass**	**0.719**
**Wing Length**	**0.754**
**Culmen Length**	**0.465**
**Head Length**	**0.768**
**Tail Length**	**0.708**
**Tarsus Length**	**0.591**

Supplementary Table 4: Model statistical outputs showing the estimates, standard error, and the lower and upper limits of the 95 % confidence interval of all model variables of each individual urban cover model relative to the body mass of the sparrows. Significant variables for each model are presented in bold text.

**Variable (Response/Control)**	**Estimate**	**Standard Error**	**Lower Limit**	**Upper Limit**	**P-value**
**Urban Overall**	-0.053	0.036	-0.123	0.017	0.0961
**specieshouse**	**-2.071**	**0.037**	**-2.144**	**-1.999**	**<2.0e-16**
**Sex1**	**-0.312**	**0.024**	**-0.359**	**-0.264**	**<2.0e-16**

Season	**-0.108**	**0.014**	**-0.135**	**-0.081**	**3.89e-13**
Temperature	**0.303**	**0.037**	**0.230**	**0.376**	**1.43e-13**
Precipitation	**0.375**	**0.045**	**0.287**	**0.464**	**1.44e-13**
Urban_Overall:specieshouse	-0.079	0.042	-0.162	0.004	0.1386
Infrastructure	**-0.250**	**0.037**	**-0.322**	**-0.177**	**3.39e-09**
specieshouse	**-2.022**	**0.037**	**-2.094**	**-1.950**	**<2.0e-16**
Sex1	**-0.310**	**0.024**	**-0.358**	**-0.263**	**<2.0e-16**
Season	**-0.107**	**0.014**	**-0.134**	**-0.080**	**5.04e-13**
Temperature	**0.263**	**0.037**	**0.190**	**0.335**	**1.03e-10**
Precipitation	**0.293**	**0.047**	**0.199**	**0.386**	**3.66e-08**
Infrastructure:specieshouse	**0.384**	**0.039**	**0.306**	**0.461**	**<2.0e-16**
Woody_Cover	**0.116**	**0.034**	**0.048**	**0.183**	**0.0051**
specieshouse	**-2.098**	**0.037**	**-2.170**	**-2.025**	**<2.0e-16**
Sex1	**-0.314**	**0.024**	**-0.361**	**-0.266**	**<2.0e-16**
Season	**-0.110**	**0.014**	**-0.137**	**-0.083**	**1.68e-13**
Temperature	**0.302**	**0.038**	**0.228**	**0.376**	**2.66e-13**
Precipitation	**0.353**	**0.045**	**0.263**	**0.443**	**4.63e-12**
Woody_Cover:specieshouse	**-0.358**	**0.044**	**-0.444**	**-0.271**	**6.94e-15**
Grass_Cover	0.061	0.032	-0.002	0.124	0.108
specieshouse	**-2.076e**	**0.037**	**-2.148**	**-2.005**	**<2.0e-16**
Sex1	**-0.314**	**0.024**	**-0.361**	**-0.266**	**<2.0e-16**
Season	**-0.111**	**0.014**	**-0.138**	**-0.084**	**1.28e-13**

**Temperature**	**0.299**	**0.037**	**0.226**	**0.372**	**3.05e-13**
**Precipitation**	**0.366**	**0.046**	**0.274**	**0.458**	**4.08e-12**
**Grass_Cover:specieshouse**	**-0.280**	**0.039**	**-0.356**	**-0.204**	**1.27e-09**

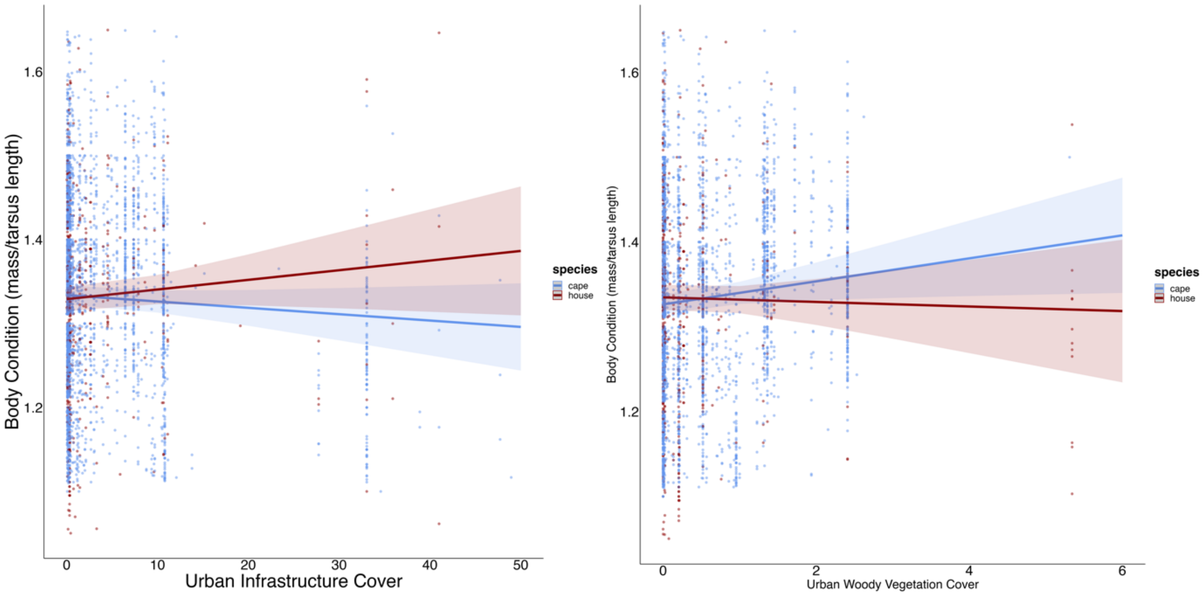

Supplementary Figure 1: The interactions of morphometric responses between the Cape (blue; n = 3, 151), *Passer melanurus*, and house (red; n = 536), *Passer domesticus*, sparrows for body condition along the different urban gradients. The interaction of body condition responses between the two sparrows a) in response to an increase in urban infrastructure cover, and b) in response to an increase in the urban woody vegetation cover (n = 3687).

<u>Supplementary Table 5: Model statistic outputs depicting the estimates, standard error, and the lower and upper limits of the 95 % confidence interval of all model variables of each individual urban cover model relative to the body condition of the sparrows. Significant variables for each model are presented in bold text.</u>

**Variable (Response/Control)**	**Estimate**	**Standard Error**	**Lower Limit**	**Upper Limit**	**P-value**
**Urban Overall**	-0.004	0.003	-0.011	0.002	0.2482

**Specieshouse**	-0.0004	0.005	-0.010	0.010	0.8437
**Sex1**	**-0.008**	**0.003**	**-0.014**	**-0.002**	**0.0190**
**Season**	-0.001	0.002	-0.005	0.002	0.5654
**Temperature**	0.003	0.003	-0.003	0.010	0.2457
**Precipitation**	**0.017**	**0.004**	**0.008**	**0.026**	**0.0002**
Urban_Overall:specieshouse	0.007	0.005	-0.002	0.016	0.1164
**Infrastructure**	-0.005	0.003	-0.01	0.002	0.1691
**specieshouse**	-0.000	0.005	-0.010	0.010	0.8511
**Sex1**	**-0.008**	**0.003**	**-0.014**	**-0.002**	**0.0188**
**Season**	-0.001	0.001	-0.005	0.002	0.5742
**Temperature**	0.003	0.004	-0.004	0.010	0.3718
**Precipitation**	**0.016**	**0.005**	**0.007**	**0.026**	**0.0006**
**Infrastructure:specieshouse**	**0.011**	**0.005**	**0.003**	**0.021**	**0.0108**
**Woody_Cover**	**0.009**	**0.004**	**0.001**	**0.017**	**0.0212**
**specieshouse**	0.003	0.005	-0.007	0.013	0.6788
**Sex1**	**-0.008**	**0.003**	**-0.014**	**-0.002**	**0.0170**
**Season**	-0.001	0.002	-0.005	0.002	0.5140
**Temperature**	0.003	0.004	-0.003	0.010	0.2652
**Precipitation**	**0.016**	**0.004**	**0.007**	**0.025**	**0.0003**
**Woody_Cover:specieshouse**	**-0.011**	**0.005**	**-0.020**	**-0.002**	**0.0168**
**Grass_Cover**	-0.009	0.005	-0.019	0.001	0.1037
**specieshouse**	0.0009	0.005	-0.009	0.012	0.9609

**Sex1**	**-0.008**	**0.003**	**-0.014**	**-0.002**	**0.0159**
**Season**	-0.001	0.002	-0.005	0.002	0.5069
**Temperature**	0.005	0.003	-0.002	0.012	0.1049
**Precipitation**	0.019	0.005	0.010	0.028	3.45e-05
**Grass_Cover:specieshouse**	0.001	0.006	-0.010	0.012	0.8270

Supplementary Table 6: Model statistic outputs depicting the estimates, standard error, and the lower and upper limits of the 95 % confidence interval of all model variables of each individual urban cover model relative to the body plan of the sparrows. Significant variables for each model are presented in bold text.

**Variable (Response/Control)**	**Estimate**	**Standard Error**	**Lower Limit**	**Upper Limit**	**P-value**
**Urban Overall**	-0.035	0.030	-0.094	0.025	0.2487
**specieshouse**	**-1.391**	**0.047**	**-1.483**	**-1.297**	**<2.0e-16**
**Sex1**	**-0.347**	**0.038**	**-0.421**	**-0.273**	**<2.0e-16**
**Season**	**-0.096**	**0.020**	**-0.134**	**-0.056**	**2.47e-06**
**Temperature**	**0.122**	**0.031**	**0.060**	**0.184**	**0.0001**
**Precipitation**	**0.174**	**0.039**	**0.096**	**0.252**	**<2.0e-16**
**Urban_Overall:specieshouse**	-0.016	0.037	-0.089	0.056	0.6612
**Infrastructure**	**-0.087**	**0.031**	**-0.149**	**-0.027**	**0.0059**
**specieshouse**	**-1.425**	**0.046**	**-1.516**	**0.015**	**<2.0e-16**
**Sex1**	**-0.342**	**0.038**	**-0.416**	**-0.014**	**<2.0e-16**
**Season**	**-0.092**	**0.020**	**-0.131**	**0.014**	**4.21e-06**
**Temperature**	**0.100**	**0.032**	**0.037**	**0.183**	**0.0019**

**Precipitation**	**0.169**	**0.042**	**-0.087**	**-0.186**	**8.10e-05**
**Infrastructure:specieshouse**	**0.109**	**0.037**	**0.036**	**0.085**	**0.0035**
**Woody_Cover**	0.037	0.039	-0.039	0.114	0.347
**specieshouse**	**-1.363**	**0.046**	**-1.453**	**-1.272**	**<2.0e-16**
**Sex1**	**-0.347**	**0.037**	**-0.421**	**-0.273**	**<2.0e-16**
**Season**	**-0.095**	**0.020**	**-0.134**	**-0.055**	**2.52e-06**
**Temperature**	**0.125**	**0.031**	**0.064**	**0.186**	**6.32e-05**
**Precipitation**	**0.188**	**0.039**	**0.111**	**0.265**	**3.35e-06**
**Woody_Cover:specieshouse**	**-0.149**	**0.041**	**-0.230**	**-0.069**	**0.0003**
**Grass_Cover**	0.076	0.049	-0.020	0.174	0.124
**specieshouse**	**-1.395**	**0.045**	**-01.482**	**-1.307**	**<2.0e-16**
**Sex1**	**-0.350**	**0.037**	**-0.424**	**-0.277**	**<2.0e-16**
**Season**	**-0.096**	**0.020**	**-0.135**	**-0.057**	**1.45e-06**
**Temperature**	**0.129**	**0.030**	**0.070**	**0.189**	**2.59e-05**
**Precipitation**	**0.201**	**0.039**	**0.124**	**0.278**	**7.69e-07**
**Grass_Cover:specieshouse**	**-0.305**	**0.054**	**-0.412**	**-0.200**	**1.95e-08**

Supplementary Table 7: Model statistic outputs depicting the estimates, standard error, and the lower and upper limits of the 95 % confidence interval of all model variables of each individual urban cover model relative to the tarsus length of the sparrows. Significant variables for each model are presented in bold text.

**Variable (Response/Control)**	**Estimate**	**Standard Error**	**Lower Limit**	**Upper Limit**	**P-value**
**Urban Overall**	**-0.103**	**0.050**	**-0.200**	**-0.004**	**0.0427**

**speciesshouse**	**-1.736**	**0.064**	**-1.861**	**-1.610**	**<2.0e-16**
**Sex1**	**-0.122**	**0.046**	**-0.212**	**-0.032**	**0.0074**
**Season**	**-0.075**	**0.026**	**-0.125**	**-0.025**	**0.0048**
**Temperature**	0.101	0.054	-0.004	0.206	0.0459
**Precipitation**	-0.116	0.068	-0.250	0.018	0.0787
**Urban_Overall:specieshouse**	-0.025	0.057	-0.136	0.086	0.6271
**Infrastructure**	**-0.186**	**0.050**	**-0.285**	**-0.088**	**0.0002**
**specieshouse**	**-1.755**	**0.063**	**-1.878**	**-1.630**	**<2.0e-16**
**Sex1**	**-0.118**	**0.046**	**-0.208**	**-0.029**	**0.0090**
**Season**	**-0.073**	**0.025**	**-0.122**	**-0.023**	**0.0065**
**Temperature**	0.051	0.056	-0.058	0.159	0.3173
**Precipitation**	**-0.158**	**0.070**	**-0.298**	**-0.019**	**0.0221**
**Infrastructure:specieshouse**	**0.130**	**0.058**	**0.017**	**0.243**	**0.0243**
**Woody_Cover**	**-0.212**	**0.064**	**-0.338**	**-0.086**	**0.0014**
**specieshouse**	**-1.738**	**0.063**	**-1.861**	**-1.613**	**<2.0e-16**
**Sex1**	**-0.123**	**0.046**	**-0.212**	**-0.034**	**0.0066**
**Season**	**-0.079**	**0.026**	**-0.129**	**-0.029**	**0.0030**
**Temperature**	**0.165**	**0.054**	**0.059**	**0.271**	**0.0018**
**Precipitation**	-0.055	0.068	-0.189	0.077	0.3716
**Woody_Cover:specieshouse**	-0.077	0.060	-0.194	0.041	0.1642
**Grass_Cover**	**0.318**	**0.079**	**0.161**	**0.475**	**4.62e-05**
**specieshouse**	**-1.730**	**0.063**	**-1.852**	**-1.608**	**<2.0e-16**

**Sex1**	**-0.121**	**0.045**	**-0.210**	**-0.032**	**0.0075**
**Season**	**-0.083**	**0.026**	**-0.133**	**-0.033**	**0.0018**
**Temperature**	0.088	0.054	-0.020	0.194	0.0920
**Precipitation**	-0.127	0.070	-0.266	0.010	0.0585
**Grass_Cover:specieshouse**	**-0.339**	**0.069**	**-0.475**	**-0.204**	**7.67e-07**

Supplementary Table 8: Model statistic outputs depicting the estimates, standard error, and the lower and upper limits of the 95 % confidence interval of all model variables of each individual urban cover model relative to the wing length of the sparrows. Significant variables for each model are presented in bold text.

**Variable (Response/Control)**	**Estimate**	**Standard Error**	**Lower Limit**	**Upper Limit**	**P-value**
**Urban Overall**	-0.028	0.045	-0.117	0.062	0.5911
**specieshouse**	**-3.785**	**0.053**	**-3.899**	**-3.672**	**<2.0e-16**
**Sex1**	**-2.607**	**0.037**	**-2.680**	**-2.534**	**<2.0e-16**
**Season**	**-0.346**	**0.021**	**-0.387**	**-0.305**	**<2.0e-16**
**Temperature**	**0.184**	**0.048**	**0.090**	**0.299**	**9.65e-05**
**Precipitation**	**0.833**	**0.058**	**0.719**	**0.948**	**<2.0e-16**
**Urban_Overall:specieshouse**	**-0.136**	**0.057**	**-0.248**	**-0.023**	**0.0138**
**Infrastructure**	**-0.014**	**0.047**	**-0.235**	**-0.050**	**0.0032**
**specieshouse**	**-3.754**	**0.058**	**-3.867**	**-3.640**	**<2.0e-16**
**Sex1**	**-2.604**	**0.037**	**-2.677**	**-2.532**	**<2.0e-16**
**Season**	**-0.349**	**0.021**	**-0.390**	**-0.308**	**<2.0e-16**
**Temperature**	**0.150**	**0.048**	**0.056**	**0.245**	**0.0014**

**Precipitation**	**0.812**	**0.061**	**0.693**	**0.932**	**<2.0e-16**
**Infrastructure:specieshouse**	**0.456**	**0.062**	**0.335**	**0.577**	**1.24e-13**
**Woody_Cover**	**0.178**	**0.046**	**0.088**	**0.268**	**5.76e-05**
**specieshouse**	**-3.782**	**0.058**	**-3.895**	**-3.668**	**<2.0e-16**
**Sex1**	**-2.608**	**0.037**	**-2.681**	**-2.536**	**<2.0e-16**
**Season**	**-0.349**	**0.021**	**-0.390**	**-0.308**	**<2.0e-16**
**Temperature**	**0.182**	**0.049**	**0.086**	**0.278**	**0.0002**
**Precipitation**	**0.808**	**0.059**	**0.692**	**0.924**	**<2.0e-16**
**Woody_Cover:specieshouse**	**-0.549**	**0.058**	**-0.664**	**-0.435**	**<2.0e-16**
**Grass_Cover**	**0.097**	**0.044**	**0.011**	**0.184**	**0.0253**
**specieshouse**	**-3.808**	**0.058**	**-3.921**	**-3.694**	**<2.0e-16**
**Sex1**	**-2.614**	**0.037**	**-2.686**	**-2.541**	**<2.0e-16**
**Season**	**-0.354**	**0.021**	**-0.395**	**-0.313**	**<2.0e-16**
**Temperature**	**0.183**	**0.048**	**0.089**	**0.278**	**0.0001**
**Precipitation**	**0.840**	**0.061**	**0.721**	**0.959**	**<2.0e-16**
**Grass_Cover:specieshouse**	**-0.684**	**0.060**	**-0.801**	**-0.567**	**<2.0e-16**

Supplementary Table 9: Model statistic outputs depicting the estimates, standard error, and the lower and upper limits of the 95 % confidence interval of all model variables of each individual urban cover model relative to the culmen length of the sparrows. Significant variables for each model are presented in bold text.

**Variable (Response/Control)**	**Estimate**	**Standard Error**	**Lower Limit**	**Upper Limit**	**P-value**
**Urban Overall**	-0.027	0.023	-0.071	0.018	0.2344

**specieshouse**	**-0.367**	**0.035**	**-0.436**	**-0.298**	**<2.0e-16**
**Sex1**	**-0.121**	**0.022**	**-0.164**	**-0.078**	**3.26e-08**
**Season**	0.017	0.012	-0.007	0.041	0.1402
**Temperature**	**0.100**	**0.023**	**0.055**	**0.146**	**1.94e-05**
**Precipitation**	**0.098**	**0.028**	**0.042**	**0.154**	**0.0005**
**Urban_Overall:specieshouse**	-0.040	0.033	-0.104	0.024	0.2440
**Infrastructure**	-0.048	0.023	-0.092	-0.003	0.0360
**specieshouse**	**-0.374**	**0.035**	**-0.443**	**-0.305**	**<2.0e-16**
**Sex1**	**-0.120**	**0.022**	**-0.164**	**-0.077**	**3.94e-08**
**Season**	0.018	0.012	-0.006	0.042	0.1157
**Temperature**	**0.090**	**0.024**	**0.043**	**0.137**	**0.0002**
**Precipitation**	**0.090**	**0.029**	**0.033**	**0.148**	**0.0019**
**Infrastructure:specieshouse**	0.035	0.033	-0.029	0.099	0.2584
**Woody_Cover**	**0.098**	**0.030**	**0.040**	**0.156**	**0.0011**
**specieshouse**	**-0.367**	**0.035**	**-0.436**	**-0.299**	**<2.0e-16**
**Sex1**	**-0.120**	**0.022**	**-0.163**	**-0.076**	**4.56e-08**
**Season**	0.016	0.012	-0.008	0.040	0.1534
**Temperature**	**0.091**	**0.024**	**0.045**	**0.137**	**0.0001**
**Precipitation**	**0.095**	**0.028**	**0.041**	**0.150**	**0.0006**
**Woody_Cover:specieshouse**	**-0.133**	**0.034**	**-0.201**	**-0.065**	**0.0001**
**Grass_Cover**	-0.044	0.041	-0.124	0.036	0.2690
**specieshouse**	**-0381**	**0.035**	**-0.450**	**-0.312**	**<2.0e-16**

**Sex1**	**-0.122**	**0.022**	**-0.165**	**-0.079**	**2.32e-08**
**Season**	0.015	0.012	-0.009	0.040	0.1744
**Temperature**	**0.107**	**0.024**	**0.060**	**0.153**	**7.66e-08**
**Precipitation**	**0.115**	**0.029**	**0.059**	**0.171**	**5.61e-05**
**Grass_Cover:specieshouse**	**-0.142**	**0.041**	**-0.224**	**-0.061**	**0.0007**

Supplementary Table 10: Model statistic outputs depicting the estimates, standard error, and the lower and upper limits of the 95 % confidence interval of all model variables of each individual urban cover model relative to the head length of the sparrows. Significant variables for each model are presented in bold text.

**Variable (Response/Control)**	**Estimate**	**Standard Error**	**Lower Limit**	**Upper Limit**	**P-value**
**Urban Overall**	**0.051**	**0.021**	**0.009**	**0.094**	**0.0222**
**specieshouse**	**-1.337**	**0.047**	**-1.430**	**-1.244**	**<2.0e-16**
**Sex1**	**-0.104**	**0.024**	**-0.151**	**-0.056**	**1.76e-05**
**Season**	0.016	0.013	-0.010	0.042	0.1676
**Temperature**	**0.083**	**0.024**	**0.035**	**0.132**	**0.0004**
**Precipitation**	-0.002	0.028	-0.057	0.053	0.9074
**Urban_Overall:specieshouse**	**-0.111**	**0.044**	**-0.197**	**-0.024**	**0.0136**
**Infrastructure**	0.021	0.027	-0.031	0.074	0.4482
**specieshouse**	**-1.351**	**0.047**	**-1.445**	**-1.260**	**<2.0e-16**
**Sex1**	**-0.102**	**0.024**	**-0.150**	**-0.055**	**2.24e-05**
**Season**	0.016	0.013	-0.009	0.042	0.1628
**Temperature**	**0.086**	**0.025**	**0.037**	**0.135**	**0.0004**

**Precipitation**	0.007	0.029	-0.051	0.064	0.8511
**Infrastructure:specieshouse**	-0.022	0.051	-0.121	0.077	0.6935
**Woody_Cover**	0.026	0.024	-0.021	0.074	0.3238
**specieshouse**	**-1.332**	**0.047**	**-1.425**	**-1.240**	**<2.0e-16**
**Sex1**	**-0.103**	**0.024**	**-0.151**	**-0.055**	**2.01e-05**
**Season**	0.016	0.013	-0.010	0.042	0.1709
**Temperature**	**0.084**	**0.025**	**0.034**	**0.134**	**0.0005**
**Precipitation**	0	0.029	-0.058	0.055	0.9632
**Woody_Cover:specieshouse**	**0.0134**	**0.040**	**-0.212**	**-0.55**	**0.0009**
**Grass_Cover**	**0.125**	**0.031**	**0.064**	**0.186**	**0.0001**
**specieshouse**	**-1.370**	**0.047**	**-1.463**	**-1.277**	**<2.0e-16**
**Sex1**	**-0.104**	**0.024**	**-0.152**	**-0.057**	**1.51e-05**
**Season**	0.018	0.015	-0.008	0.044	0.1270
**Temperature**	**0.067**	**0.025**	**0.019**	**0.116**	**0.0038**
**Precipitation**	-0.022	0.028	-0.079	0.034	0.4200
**Grass_Cover:specieshouse**	**-0.242**	**0.064**	**-0.367**	**-0.116**	**0.0002**

Supplementary Table 11: Model statistic outputs depicting the estimates, standard error, and the lower and upper limits of the 95 % confidence interval of all model variables of each individual urban cover model relative to the tail length of the sparrows. Significant variables for each model are presented in bold text.

**Variable (Response/Control)**	**Estimate**	**Standard Error**	**Lower Limit**	**Upper Limit**	**P-value**
**Urban Overall**	-0.015	0.065	-0.143	0.112	0.8594

**specieshouse**	**-5.096**	**0.110**	**-5.313**	**-4.881**	**<2.0e-16**
**Sex1**	**-2.007**	**0.068**	**-2.139**	**-1.874**	**<2.0e-16**
**Season**	**-0.460**	**0.038**	**-0.534**	**-0.386**	**<2.0e-16**
**Temperature**	0.137	0.070	-0.001	0.276	0.0532
**Precipitation**	**0.719**	**0.083**	**0.557**	**0.882**	**<2.0e-16**
**Urban_Overall:specieshouse**	0.120	0.087	-0.052	-0.291	0.2261
**Infrastructure**	**-0.127**	**0.064**	**-0.253**	**-0.002**	**0.0513**
**specieshouse**	**-5.148**	**0.110**	**-5.365**	**-4.793**	**<2.0e-16**
**Sex1**	**-2.006**	**0.067**	**-2.138**	**-1.874**	**<2.0e-16**
**Season**	**-0.463**	**0.038**	**-0.537**	**-0.389**	**<2.0e-16**
**Temperature**	0.113	0.071	-0.027	0.253	0.1139
**Precipitation**	**0.692**	**0.087**	**0.521**	**0.863**	**1.79e-14**
**Infrastructure:specieshouse**	**0.355**	**0.081**	**0.196**	**0.514**	**1.35e-05**
**Woody_Cover**	0.128	0.087	-0.043	0.298	0.1188
**specieshouse**	**-5.007**	**0.109**	**-5.222**	**-4.793**	**<2.0e-16**
**Sex1**	**-2.010**	**0.067**	**-2.143**	**-1.878**	**<2.0e-16**
**Season**	**-0.463**	**0.038**	**-0.537**	**-0.389**	**<2.0e-16**
**Temperature**	0.124	0.071	-0.016	0.266	0.0848
**Precipitation**	**0.695**	**0.083**	**0.532**	**0.859**	**1.10e-15**
**Woody_Cover:specieshouse**	**-0.410**	**0.100**	**-0.605**	**-0.215**	**1.32e-05**
**Grass_Cover**	**0.212**	**0.094**	**0.029**	**0.396**	**0.0147**
**specieshouse**	**-5.133**	**0.109**	**-5.349**	**-4.919**	**<2.0e-16**

**Sex1**	**-2.013**	**0.067**	**-2.145**	**-1.881**	**<2.0e-16**
**Season**	**-0.468**	**0.038**	**-0.541**	**-0.393**	**<2.0e-16**
**Temperature**	0.120	0.070	-0.019	0.260	0.0968
**Precipitation**	**0.688**	**0.084**	**0.524**	**0.852**	**3.43e-15**
**Grass_Cover:specieshouse**	**-0.680**	**0.139**	**-0.951**	**-0.408**	**4.44e-07**
